# A Novel, Functional and Replicable Risk Gene Region for Alcohol Dependence Identified by Genome-Wide Association Study

**DOI:** 10.1371/journal.pone.0026726

**Published:** 2011-11-07

**Authors:** Lingjun Zuo, Clarence K. Zhang, Fei Wang, Chiang-Shan R. Li, Hongyu Zhao, Lingeng Lu, Xiang-Yang Zhang, Lin Lu, Heping Zhang, Fengyu Zhang, John H. Krystal, Xingguang Luo

**Affiliations:** 1 Department of Psychiatry, Yale University School of Medicine, New Haven, Connecticut, United States of America; 2 Veterans Affairs Connecticut Healthcare System, West Haven, Connecticut, United States of America; 3 Department of Epidemiology and Public Health, Yale University School of Medicine, New Haven, Connecticut, United States of America; 4 Department of Psychiatry, The First Affiliated Hospital, China Medical University, Shenyang, China; 5 Menninger Department of Psychiatry and Behavioral Sciences, Baylor College of Medicine, Houston, Texas, United States of America; 6 National Institute on Drug Dependence, Peking University, Beijing, China; 7 Gene, Cognition and Psychosis Program, National Institute of Mental Health, National Institutes of Health, Bethesda, Maryland, United States of America; National cancer Institute, United States of America

## Abstract

Several genome-wide association studies (GWASs) reported tens of risk genes for alcohol dependence, but most of them have not been replicated or confirmed by functional studies. The present study used a GWAS to search for novel, functional and replicable risk gene regions for alcohol dependence. Associations of all top-ranked SNPs identified in a discovery sample of 681 African-American (AA) cases with alcohol dependence and 508 AA controls were retested in a primary replication sample of 1,409 European-American (EA) cases and 1,518 EA controls. The replicable associations were then subjected to secondary replication in a sample of 6,438 Australian family subjects. A functional expression quantitative trait locus (eQTL) analysis of these replicable risk SNPs was followed-up in order to explore their *cis-*acting regulatory effects on gene expression. We found that within a 90 Mb region around *PHF3-PTP4A1* locus in AAs, a linkage disequilibrium (LD) block in *PHF3-PTP4A1* formed the only peak associated with alcohol dependence at p<10^−4^. Within this block, 30 SNPs associated with alcohol dependence in AAs (1.6×10^−5^≤p≤0.050) were replicated in EAs (1.3×10^−3^≤p≤0.038), and 18 of them were also replicated in Australians (1.8×10^−3^≤p≤0.048). Most of these risk SNPs had strong *cis-*acting regulatory effects on *PHF3-PTP4A1* mRNA expression across three HapMap samples. The distributions of −log(p) values for association and functional signals throughout this LD block were highly consistent across AAs, EAs, Australians and three HapMap samples. We conclude that the *PHF3-PTP4A1* region appears to harbor a causal locus for alcohol dependence, and proteins encoded by *PHF3* and/or *PTP4A1* might play a functional role in the disorder.

## Introduction

Alcohol dependence is a common, highly familial disorder that is a leading cause of morbidity and premature death. It results in serious medical, legal, social and psychiatric problems and influences many facets of American society. It affects 4 to 5% of the United States population at any given time, with a lifetime prevalence of 12.5% [1], [2]. Family, twin and adoption studies have demonstrated that genetic factors constitute a significant cause for alcohol dependence. A large number of risk loci have been reported for alcohol dependence (AD) by candidate gene approach. Several genome-wide association studies (GWASs) [3], [4], [5], [6], [7] have also reported tens of risk loci for alcohol dependence and alcohol consumption (summarized by Zuo et al. [3]). However, most GWAS findings have not been replicated in independent samples and confirmed by functional studies.

In the present study, we reanalyzed the data sets of the Study of Addiction Genetics and Environment (SAGE), the Collaborative Study on the Genetics of Alcoholism (COGA) and the Australian family study of alcohol use disorder (OZ-ALC). Using the following analytic strategies, we expected to discover the novel (i.e., previously unimplicated) risk loci for alcohol dependence. First, we combined SAGE and COGA datasets to increase the sample sizes and power (with site-to-site variation and sample overlapping being considered), which may be able to detect some novel risk loci missed in previous studies. Second, we set AAs as the discovery sample. The top-ranked SNP list in AAs would be different from those in the previous studies that used EAs, Germans or Australians as the discovery sample. Third, we used replication and confirmation design to reduce the chance of false positive findings, and thus increase α level, which may be able to detect some novel risk loci missed in previous studies due to too conservative Bonferroni correction. Fourth, we completely separated EAs and AAs in the analysis to increase the population homogeneity, and controlled for admixture effects in the association tests. Fifth, we used EAs and Australians as replication samples, and then used different samples with distinct ethnicity to detect eQTL signals, as a confirmation of variant functions to the discovery association findings. Although using distinct samples in one study might increase the false negative rates due to sample heterogeneity, replication in distinct samples does make the false positive findings less likely. Replicable findings in distinct populations would be more generalizable to more other populations, and would be more likely to appear on the causal variants. Sixth, we applied innovative definition of replication. The primary target of investigation in the current study was not the top-ranked SNPs in the discovery sample as previous GWASs, but rather the replicable risk regions. This idea was similar to that in a prior study [4]. In the replicable risk regions, there should be not only many individual markers replicable between the discovery and replication samples, but the overall distributions of association signals and functional signals throughout the whole region should also be consistent across the discovery, replication and confirmation samples (see rationales in Materials and Methods S1). Such important regions have not been reported in previous GWASs of alcohol dependence.

## Materials and Methods

### Subjects

A total of 10,554 subjects underwent gene-disease association analysis, including (i) a discovery sample of 681 African-American (AA) cases with alcoholism (37.2% females; 40.3±7.8 years) and 508 AA controls (66.7% females; 39.6±8.6 years), (ii) a primary replication sample of 1,409 European-American (EA) cases (37.3% females; 38.3±10.2 years) and 1,518 EA controls (70.7% females; 39.4±10.4 years), and (iii) a secondary replication sample of 6,438 Australian family subjects (51.9% females; 46.0±10.0 years; 1,645 affected subjects including 625 females). AA and EA samples came from merged SAGE (dbGaP study accession phs000092.v1.p1) and COGA (dbGaP: phs000125.v1.p1) datasets [5], [6], and Australian sample was OZ-ALC (dbGaP: phs000181.v1.p1) dataset [7]. These datasets were originally collected mainly for study of alcoholism. All Australian subjects were of European ancestry. Affected subjects met lifetime DSM-IV criteria for alcohol dependence [8], and Australian subjects were also measured for alcohol consumption by a quantitative scale. Controls were defined as individuals who had been exposed to alcohol (and possibly to other drugs) at sufficient amounts for a sufficient time, but had never become addicted to alcohol or other illicit substances (lifetime diagnoses). This criterion for controls took into account the confounding effects from an environmental factor, i.e., drinking. In contrast to general controls who had never used substances, our controls reduced the potential false negative rates, because a proportion of general controls might still have a risk to develop to alcohol dependence when drinking. Additionally, controls were also screened to exclude individuals with major axis I disorders, including schizophrenia, mood disorders, and anxiety disorders. More detailed demographic information is available in Materials and Methods S1 or elsewhere [3], [5], [6], [9]. AA and EA samples were genotyped on the Illumina Human 1 M beadchip and Australian sample was genotyped on the Illumina CNV370v1 beadchip.

### Ethics Statement

All subjects gave written informed consent to participate in protocols approved by the relevant institutional review boards (IRBs). All subjects were de-identified in this study and the study was approved by Yale IRB.

### Imputation

CNV370 beadchip has only one-sixth of markers overlapping with Human1M beadchip. To know if the risk markers identified in AAs and EAs (Human1M) could be replicated in Australians (CNV370), we imputed the genotype data in Australians to fill in the missing markers and then performed association tests. First, we pre-phased the original genotype data 5 Mb around the risk genes of interest in Australians. Second, we used 1,000 Genome Project and HapMap 3 CEU datasets as reference panels to impute the missing genotypes in this 5 Mb region by the program IMPUTE2 [10]. This program uses a Markov Chain Monte Carlo (MCMC) algorithm to derive full posterior probabilities of genotypes of each SNP (burnin = 10, iteration = 30, k = 80 and Ne = 11,500). If the probability of one of the three genotypes of a SNP was over the threshold of 0.95, the genotypes of this SNP were then expressed as a corresponding allele pair for the following association analysis; otherwise, they were treated as missing genotypes. For SNPs that were directly genotyped, we used the direct genotypes rather than the imputed data. The imputed genotype data in Australians were checked for Mendelian errors by the program PEDCHECK [11].

### Association analysis

Before statistical analysis, we cleaned the phenotype data first and then the genotype data. This cleaning process yielded 805,814 SNPs in EAs, 895,714 SNPs in AAs and 300,839 SNPs in Australians. [Detailed cleaning steps were described previously [3]].

**Figure 1:**
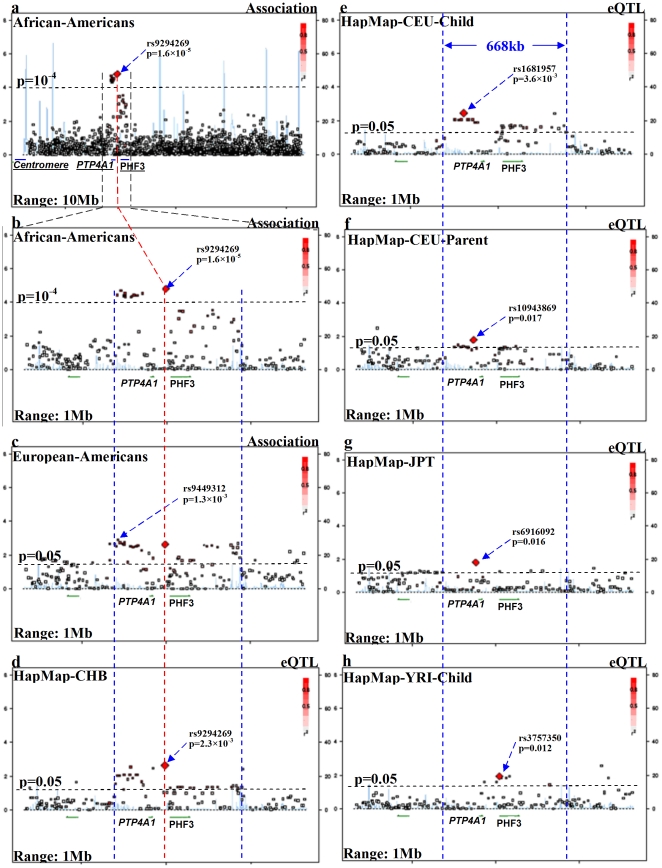
Regional association and eQTL plots around PHF3-PTP4A1 region. [Left Y-axis corresponds to −log(p) value; right Y-axis corresponds to recombination rates; quantitative color gradient corresponds to r^2^; red squares represent peak SNPs. (a) regional association plot in AAs for a 10 Mb region surrounding the peak association SNP (rs9294269) in PHF3-PTP4A1; (b, c) regional association plots in AAs or EAs for a 1 Mb region surrounding the peak association SNP (rs9294269) in PHF3-PTP4A1; (d–h) regional eQTL plots in HapMap populations for a 1 MB region surrounding rs9294269; (i) LD map for all available markers for a region surrounding rs9294269 in EAs (Illumina Human1M beadchip), in which red bars represent the peak SNPs in each population].

- Genome-wide association tests in AA discovery sample: The allele and genotype frequencies were compared between cases and controls in AAs using genome-wide logistic regression analysis implemented in the program PLINK [12]. Diagnosis served as the dependent variable, alleles or genotypes served as the independent variables, and ancestry proportions (to control for admixture effects), sex, and age served as covariates. Ancestry proportions of each individual were estimated from 3,172 completely independent markers [3]. The top-ranked SNPs (p<10^−4^) were also tested by Fisher's exact tests without controlling for admixture effects. The p-values derived from these analyses are illustrated in **Figure S1** and the top 5 SNPs are listed in **Table S1**.
- Association tests in the primary EA replication sample: Associations between the above top-ranked SNPs (p<10^−4^) and alcohol dependence were tested using logistic regression analysis (with ancestry proportions, sex and age as covariates) and Fisher's exact test (without covariates) in EAs, to identify risk genes (i.e., Plant HomeoDomain (PHD) finger protein 3 gene - protein tyrosine phosphatase type IVA gene, member 1 (*PHF3-PTP4A1*) here) that were enriched with replicable markers. Then, associations between alcohol dependence and all nominally significant SNPs (p<0.05 in AAs) in *PHF3-PTP4A1* were retested in EAs. The associations that were replicated across AAs and EAs are shown in **Table S1** and **Figure 1**. Meta-analysis was performed to derive the combined p values between AAs and EAs.
- Family-based association tests in the secondary Australian family replication sample: Associations between alcohol dependence and the replicable risk SNPs in *PHF3-PTP4A1* (**Table 1**) identified between AAs and EAs were retested in Australians using a family-based association test implemented in PLINK [12]. Meta-analysis was performed to derive the combined p values between EAs and Australians.

**Table 1:** P-values for replicable association and eQTL signals.

		Associations						*Cis* -eQTL in HapMap samples		
		AAs		EAs		Australians		p-value		
Gene	SNP	OR	p-value	OR	p-value	OR	p-value	CEU-Child	CEU-parent	CHB
PTP4A1	rs9449291	1.54	3.8×10^−5^	0.85	3.1×10^−3^	0.79	0.048	-	-	0.028
PTP4A1	rs9449312	1.18	0.050	0.84	1.3×10^−3^	0.79	0.047	-	-	9.6×10^−3^
PTP4A1	rs6942342	1.56	2.0×10^−5^	0.85	1.8×10^−3^	0.81	0.062	-	-	9.6×10^−3^
PTP4A1	rs9353016	1.54	4.1×10^−5^	0.85	2.2×10^−3^	0.85	-	-	-	-
PTP4A1	rs4299811	1.54	3.0×10^−5^	0.85	2.5×10^−3^	0.83	-	8.7×10^−3^	0.043	8.3×10^−3^
PTP4A1	rs4557499	1.52	4.5×10^−5^	0.85	2.2×10^−3^	0.83	-	-	-	-
PTP4A1	rs2758259	1.52	5.0×10^−5^	0.85	1.8×10^−3^	0.83	-	8.7×10^−3^	0.045	8.3×10^−3^
PTP4A1	rs1744134	0.82	0.032	1.14	0.027	1.08	-	9.2×10^−3^	0.034	0.016
PTP4A1	rs1744140	1.52	4.9×10^−5^	0.85	3.0×10^−3^	0.85	-	8.7×10^−3^	0.045	8.6×10^−3^
PTP4A1	rs2984458	1.52	4.4×10^−5^	0.85	3.0×10^−3^	0.85	-	8.7×10^−3^	0.045	2.9×10^−3^
PTP4A1	rs1681957	1.54	3.8×10^−5^	0.85	3.3×10^−3^	0.85	-	3.6×10^−3^	0.040	0.013
PTP4A1	rs1197905	1.54	3.8×10^−5^	0.85	3.0×10^−3^	0.85	-	8.7×10^−3^	0.045	8.3×10^−3^
PTP4A1	rs2622274	1.27	5.6×10^−3^	0.85	2.7×10^−3^	0.85	-	8.7×10^−3^	0.047	8.3×10^−3^
PTP4A1	rs1322416	1.54	3.0×10^−5^	0.86	5.2×10^−3^	0.86	-	8.7×10^−3^	-	0.022
PHF3	rs9294269	1.56	1.6×10^−5^	1.17	2.4×10^−3^	-	-	0.051	-	2.3×10^−3^
PHF3	rs6932538	1.39	9.4×10^−4^	0.88	0.016	0.76	0.015	0.027	-	0.045
PHF3	rs10485358	1.45	3.2×10^−4^	0.88	0.014	0.78	0.023	0.018	-	0.045
PHF3	rs10755432	1.43	3.4×10^−4^	0.88	0.022	0.76	0.019	0.021	-	0.045
PHF3	rs1057530	1.30	5.7×10^−3^	0.88	0.022	0.76	0.019	0.027	-	0.049
PHF3	rs12205302	1.33	2.6×10^−3^	0.88	0.021	0.74	8.9×10^−3^	0.027	-	0.048
PHF3	rs319924	1.41	6.4×10^−4^	0.85	3.0×10^−3^	0.71	2.9×10^−3^	0.024	-	0.043
PHF3	rs319920	1.43	2.9×10^−4^	0.85	2.7×10^−3^	0.71	3.5×10^−3^	-	-	0.045
PHF3	rs756274	1.34	5.0×10^−4^	0.89	0.038	0.80	0.041	-	-	-
PHF3	rs6921058	1.37	8.0×10^−4^	0.85	3.4×10^−3^	0.71	4.1×10^−3^	0.026	-	0.045
PHF3	rs12205984	1.33	1.6×10^−3^	0.85	2.6×10^−3^	0.71	3.1×10^−3^	0.050	-	0.045
PHF3	rs321498	1.22	0.019	0.86	7.7×10^−3^	0.74	0.012	-	-	0.037
PHF3	rs321494	1.39	1.1×10^−3^	0.85	3.5×10^−3^	0.70	2.7×10^−3^	-	-	-
PHF3	rs729291	1.30	5.2×10^−3^	0.85	3.7×10^−3^	0.69	1.8×10^−3^	-	-	0.051
PHF3	rs1482451	0.81	0.017	1.19	2.0×10^−3^	1.32	0.016	-	-	-
PHF3	rs3003672	0.79	0.016	2.86	7.9×10^−3^	3.23	6.1×10^−3^	-	-	-

*SNPs are ordered by chromosome positions (see Table S4). All SNPs were in Hardy-Weinberg Equilibrium (HWE) (p>0.05) in both cases and controls for both AAs and EAs. SNPs underlined in Australian group were imputed markers.*

*OR corresponds to the minor alleles in AAs listed in Table S4. Region-wide α was set at 0.01.*

### *Cis-*acting genetic regulation of expression analysis

To examine relationships between genetic variants and local gene expression levels in lymphoblastoid cell lines, we performed *cis*-acting expression of quantitative locus (*cis*-eQTL) analysis. These relationships included those between all replicable risk SNPs in *PHF3* and *PHF3* mRNA expression levels, and those between all replicable risk SNPs in *PTP4A1* and *PTP4A1* mRNA expression levels. Expression data of 14,925 transcripts (14,072 genes) in 270 unrelated HapMap individuals from six populations [Utah residents with Northern and Western European ancestry from the CEPH collection (CEU)-Children, CEU-Parent, Han Chinese in Beijing (CHB), Japanese in Tokyo (JPT), Yoruba in Ibadan (YRI)-Children and YRI-Parent] were evaluated [13]. Differences in the distribution of mRNA expression levels between SNP genotypes were compared using a Wilcoxon-type trend test. P-values less than 0.05 were listed in **Table 1** and plotted in **Figure 1**. Additionally, effects of SNPs 1 Mb surrounding the association peak SNP (rs9294269) were illustrated in **Figures 1D–H**.

### Correction for multiple testing

The AA discovery sample was genotyped for one million SNPs. The association results could be corrected for one million tests (α = 5×10^−8^) to prevent from false positive findings. However, this correction is overly conservative, because these 1 M markers are not completely independent. Instead, in the present study, we used multiple samples to replicate and confirm the discovery findings, in order to reduce the chance of false positive findings and increase the α level from 5×10^−8^. First, we used EAs and Australians, the most genetically distinct populations from AAs in the world, as replication groups for association analysis. This would make the replicable findings more generalizable to more other populations. Second, we aimed to detect replicable regions that were enriched with many, not a single, risk markers, which reduced the chance of false positive association findings too. Third, functional analysis as confirmation of association analysis further reduced the chance of false positive findings. Additionally, functional analysis in multiple populations with distinct ethnicity, which were also different from the populations for association analysis, would make the findings more generalizable too. Fourth, the distributions of −log(P) values across the discovery, replication, and confirmation samples were compared for the similarity using Pearson correlation analysis (see rationale in Materials and Methods S1). The consistency between them would significantly reduce the chance of false positive findings. Additionally, our analyses followed a fixed procedure (Materials and Methods S1) step-by-step, which reduced multiple testing. Therefore, α in the discovery sample was not necessary to be corrected for one million of times if an association was replicated.

Furthermore, only when a discovery finding was replicated and confirmed by multiple groups, it was taken as “significant” in the present study. For these replicable findings, a region-wide correction might be sufficient. Five independent markers, which were the effective number capturing the information content of all 30 replicable risk markers in whole *PHF3-PTP4A1* region (**Table 1**), were predicted by the program SNPSpD [14]. Thus, a region-wide corrected α could be set at 0.01 ( = 0.05/5) for those replicable findings.

### Transcriptome-wide expression correlation analysis

The expression data of 14,925 transcripts in 93 autopsy-collected frontal cortical brain tissue samples were evaluated using Affymetrix Human ST 1.0 exon arrays. These data were obtained from a research study [15] at Duke University. These individuals included 55 males and 38 females, from 34 to 104 years old with an average of 74±16 years. The postmortem intervals, i.e., the time from death to brain tissue collection, were 1.2–46 hours with an average of 14.3±9.5 hours. These individuals had no defined neuropsychiatric condition. Correlations between expression of *PHF3-PTP4A1* transcript and expression of other genes across transcriptome in these individuals were tested (**Table S2**). α was set at 3.4×10^−6^ ( = 0.05/14,925).

## Results

There were a total of 114 SNPs in 79 genes that were marginally (p<10^−4^) associated with alcohol dependence in the AA discovery sample (data available on request). The p values from the allelewise and genotypewise association analyses of the five top-ranked SNPs before and after controlling for admixture effects are listed in **Table S1**. Among these top-ranked SNPs, 22 SNPs (19.3%) in 10 genes were replicable in EAs (**Table S3**). Among these 10 genes, only *PHF3-PTP4A1* region was enriched with 12 replicable top-ranked SNPs (**Table S3**).

Testing all available SNPs (n = 131) in the *PHF3-PTP4A1* region in AAs, we found 38 SNPs that were nominally associated (1.6×10^−5^≤p≤0.050) with alcohol dependence, among which, 28 survived region-wide correction for multiple testing (α = 0.01). Testing these 38 SNPs in EAs, we found 30 in one LD block (D′>0.9; **Figure 1**) that were well replicated in EAs (1.3×10^−3^≤p≤0.038), and 23 of them that survived region-wide correction (α = 0.01) (**Table 1**). Testing all of these 30 SNPs in Australians, we found 18 SNPs that were replicable in this sample (1.8×10^−3^≤p≤0.048), and 9 of them that survived region-wide correction (α = 0.01) (**Table 1**). Interestingly, 29 risk SNPs had same direction of gene effects on alcohol dependence between EAs and Australians, but had opposite directions of effects between EAs and AAs (**Table 1**). Meta-analysis showed that these gene effects became less significant when combined AAs and EAs, but became a little more significant when combined EAs and Australians (data not shown). In spite of this, all risk alleles (OR>1; **Table 1**) of these 29 SNPs (except for rs1744134, rs1482451 and rs3003672) were the minor alleles (*f*<0.5) in both AAs and EAs (**Table S4**). Additionally, sex, age and admixture effects did not significantly affect our results (data not shown).

*Cis-*eQTL analysis showed that 24 of the 30 replicable risk SNPs had significant *cis-*acting regulatory effects on *PHF3-PTP4A1* mRNA expression level in at least one of HapMap CEU-Children, CEU-Parent and CHB populations (**Table 1** **;** **Figure 1D–F**), and 12 of them survived region-wide correction (α = 0.01). *PHF3-PTP4A1* was enriched with many other functional signals across five HapMap populations (**Figure 1**), although these functional SNPs in JPT and YRI-Parent were not exactly, but in high LD with, those replicable risk SNPs in the AA discovery sample (**Figure 1G–H**).

The LD block of *PHF3-PTP4A1* containing the association signals overlapped extensively across AAs, EAs and Australians (**Figure 1B and 1C**; **Table 2**). The LD block that was enriched with functional signals across HapMap CEU-Children, CEU-Parent and CHB populations overlapped extensively with the region that had significant association signals across AAs, EAs and Australians (**Figure 1B–C and 1D–F**). The distributions of −log(p) values for association and functional signals across AA, EA, Australians, CHB and CEU-Children populations were highly consistent (Pearson correlation coefficient r≥0.465 with 2.5×10^−21^≤p≤4.0×10^−4^), and were negatively correlated with that in YRI-Children (r≤−0.407; 5.7×10^−6^≤p≤9.2×10^−4^; **Table 2** and **Figure 1**).

**Table 2:** Correlation of −log(p) value distributions of gene-disease associations and gene expression between different populations.

		Pearson correlation coefficients (r)								
	Populations	AA	EA	Australians	CHB	CEUchild	CEUparent	JPT	YRIchild	YRIparent
p-values	AA		0.812	0.335	0.749	0.549	0.029	−0.157	−0.530	0.007
	EA	2.5×10^−21^		0.545	0.802	0.465	0.016	−0.307	−0.407	0.022
	Australians	0.007	3.8×10^−6^		0.177	0.067	−0.456	−0.233	−0.080	0.211
	CHB	1.1×10^−12^	1.6×10^−15^	-		0.004	0.233	−0.190	−0.585	−0.107
	CEUchild	1.7×10^−5^	4.0×10^−4^	-	-		0.459	−0.105	−0.058	0.039
	CEUparent	-	-	0.001	-	4.8×10^−4^		−0.307	0.114	−0.192
	JPT	-	0.012	-	-	-	0.030		−0.093	0.346
	YRIchild	5.7×10^−6^	0.001	-	2.7×10^−6^	-	-	-		−0.043
	YRIparent	-	-	-	-	-	-	0.008	-	

*r, Pearson correlation coefficient; p, p-values for pairwise correlations; “-”, p>0.05.*

In the AA discovery sample, within the 25 Mb region around the peak association SNP (rs9294269; p = 1.6×10^−5^), this risk LD block formed the only peak that had association signals significant at a p<10^−3^; within the 90 Mb region around this SNP, this risk LD block was the only peak that had association signals significant at a p<10^−4^ (see **Figure 1A**, which depicts 10 Mb of this interval). In the EA replication sample, within the 10 Mb region around the peak SNP (rs9449312; p = 1.3×10^−3^), this risk LD block was the only peak that had association signals significant at a p<1.5×10^−3^ (see **Figure 1C**, which depicts 1 Mb of this interval). Additionally, within 1 Mb range, the most significant functional SNPs in HapMap CHB (rs9294269; p = 0.0023; **Figure 1D**), CEU-Child (rs1681957; p = 0.0036; **Figure 1E**), and JPT (rs6916092; p = 0.016; **Figure 1G**), and the second most significant functional SNPs in CEU-Parent (rs10943869; p = 0.017; **Figure 1F**) and YRI-Child (rs3757350; p = 0.012; **Figure 1H**) were all located in *PHF3-PTP4A1*. The peak SNPs among each of these populations were in high LD (D′>0.9); especially, the peak SNP in CHB (rs9294269) was exactly the same peak SNP in AAs (**Figure 1B** vs. **1D**). The more closely the peak SNPs were located (**Figure 1I**), the correlations between the distributions of −log(p) values across whole region were more significant (**Table 2**), which suggested that the peak SNP captured most information of the whole distribution across that region. The more significant those correlations were, the more consistent (replicable) between populations the risk regions would be. Thus, the distance between peak SNPs reflected the strength of replicability of association or function signals between populations.

Finally, transcriptome-wide expression correlation analysis showed that expression of *PHF3* and *PTP4A1* transcripts in brain was significantly correlated with expression of many alcoholism-related genes (**Table S2**) (although some associations between these genes and alcoholism have not yet been well replicated so far). Interestingly, many of these genes were those top-ranked genes identified by previous GWASs on alcoholism [5], [6], [16], including the GWAS in German sample that was different from the datasets we used. These top-ranked gene included *NRD1*, *PDE4B*, *OLFM3*, *NXPH2*, *PECR*, *PPARG*, *SH3BP5*, *BBX*, *PCDH7*, *LOC91431*, *IPO11*, *CAST*, *ERAP1*, *PPP2R2B*, *FAM44B*, *ANKS1A*, *EPHA7*, *NAP1L4*, *CARS*, *CCDC41*, *PCDH9*, *CDH8* and *CDH13* (see **Table S2**). Additionally, some genes co-expressed with *PHF3* and *PTP4A1* were from the dopaminergic (*DRD2*, *DRD4* and *TH*) [17], serotoninergic (*HTR2A*, *HTR3B* and *SLC6A4*) [18], GABAergic (*GABRA1*, *GABRA2*, *GABRB1*, *GABRB2* and *GABRG2*) [19], glutamatergic (*GAD1*) [6], histaminergic (*HNMT*) [20] and endocannabinoid (*CNR1*) [21] systems (α = 3.4×10^−6^).

## Discussion

In the present study, when merging 480 COGA subjects into SAGE sample, we got highly similar results to previous studies that used SAGE sample alone [5], [22]. The top-ranked risk SNPs (p<10^−5^) in EAs, AAs, and AAs+EAs in those previous studies [5], [22] were confirmed by our analysis (presented previously [3]). Similarly, many top-ranked risk SNPs (**Table S1**) in the present study were also listed as top-ranked genes previously. However, these top-ranked genes have not yet been replicated independently and confirmed by functional studies before.

In the present study, using new analytic strategy and integrating evidence from the functional analysis, we identified a risk region for alcohol dependence (i.e., *PHF3-PTP4A1* locus) that was missed previously. This region was enriched with functional genetic SNPs that had replicable associations with alcohol dependence. This important risk region was not reported previously, because most of the risk SNPs in it had p-values between 10^−5^ and 10^−3^ that were out of the top-ranked risk SNP list (p<10^−5^) in previous GWASs. Such p values were reasonable for alcohol dependence, because the effect sizes of individual loci for this complex trait had to be small. We used a replication design to reduce the false positive rate and increase the significance threshold (α) from 5×10^−8^, and thus discovered this risk region.

*PHF3-PTP4A1* region was enriched with 30 replicable risk SNPs for alcohol dependence in two kinds of genetically distinct populations, i.e., AAs and EAs. Twenty-six of these replicable risk SNPs were found to be functional by expression data obtained across multiple HapMap populations. All risk SNPs were in one LD block around the association peak SNP (i.e., rs9294269 in *PHF3* in AAs). This risk LD block overlapped extensively across AAs, EAs, Australians and three HapMap populations, and the association or functional peak SNPs in each of these populations were in high LD with each other. In a word, the association and functional signals in this LD block were highly consistent across six samples.

These findings suggested that the *PHF3-PTP4A1* region might harbor a causal locus and that the proteins encoded by *PHF3* and *PTP4A1* might contribute to the vulnerability to alcohol dependence. First, the risk LD block in the region of *PHF3-PTP4A1* formed the only association peak within a 90 Mb region in AAs (threshold p = 10^−4^) and within a 10 Mb region in EAs (threshold p = 1.5×10^−3^). It is, thus, highly likely that the putative causal locus for alcohol dependence was located within this *PHF3-PTP4A1* LD block. We speculated that there might be only one causal locus in this region, and all risk SNPs might be in LD with this putative causal locus and, thus, presented association signals. If there were ≥2 independent causal loci, the risk markers in LD with respective causal loci would be located in ≥2 independent risk LD blocks, which were not observed in the present study. Second, most replicable risk SNPs in this block had strong *cis-*acting regulatory effects on *PHF3-PTP4A1* mRNA expression. This increased the possibility that *PHF3-PTP4A1 per se* played a direct functional role in the disorder. Third, many *PHF3-PTP4A1* SNPs had significant (in *PHF3*) or slight (in *PTP4A1*) potential for altering the secondary RNA structure (predicted by MFOLD [23]) (**Table S4**), providing additional evidence in support of the hypothesis that *PHF3-PTPA41 per se* contributed to alcohol dependence. Fourth, distributions of −log(P) values for gene-disease associations and for gene-expression associations were highly consistent across at least six populations. This might suggest that the majority of the functions of *PHF3-PTP4A1* contributed to the risk for alcohol dependence, and that the regulatory pathway via which these SNPs caused alcohol dependence might be related to the PHF3 and PTP4A1 proteins *per se*. Taken together, these findings strongly supported the hypothesis that *PHF3-PTP4A1* harbored a causal locus for alcohol dependence.

It is well-known that the gene expression is tissue-specific. In another word, consistent findings between lymphoblastoid cell lines and brain tissues are rare, but inconsistent findings between them are common. Suppose the alcoholism-associated markers have positive *cis-*eQTL signals in the brain, the chance of these markers happening to have negative *cis-*eQTL signals (i.e., false negative rate) in the lymphoblastoid cell lines could be common; but the chance of these markers happening to have positive *cis-*eQTL signals (i.e., false positive rate) in the lymphoblastoid cell lines is rare; and the chance of these markers happening to have distributions highly consistent between *cis-*eQTL signals in the lymphoblastoid cell lines and gene-disease association signals across different samples should be extremely rare. That is, using lymphoblastoid cell lines for *cis-*eQTL analysis of brain disorder-related markers might increase the false negative rates due to the relatively poor conservation in *cis-*eQTLs between cell lines and brain tissue samples, but it should not significantly increase the false positive rates. In the present study, (1) we detected positive *cis-*eQTL signals in lymphoblastoid cell lines across multiple populations, (2) these markers were alcoholism-associated, and (3) the distributions of these *cis-*eQTL signals matched the distribution of the alcoholism-gene association signals. We believed that these findings might be highly likely to be truly positive, and strongly suggested that these markers might have positive *cis-*eQTL signals in the brain too. Independent validation of the *cis-*eQTL analysis in the brain tissues is warranted in the follow-up study to test our hypothesis.

*PHF3* and *PTP4A1* might also influence alcohol dependence by interacting with other genes. Expression of *PHF3* and *PTP4A1* transcripts was significantly correlated with expression of many alcoholism-related genes in brain, including those in the dopaminergic, serotoninergic, GABAergic, glutamatergic, histaminergic and endocannabinoid systems [6], [17], [18], [19], [20], [21]. These findings suggested that *PHF3* and *PTP4A1* might also be implicated in alcohol dependence via the classical neurotransmission systems or metabolic pathways.

It is worth noting that the putative causal locus within the *PHF3-PTP4A1* region may not be identical to the risk markers implicated in the current study, and therefore, may need to be identified by sequencing. First, none of the risk SNPs presented here were non-synonymous. Rather, they appear to have implications for risk and function by virtue of their being in LD with a putative causal locus and/or due to their location in regulatory regions (e.g., enhancer elements) that may in turn regulate transcription of the causal locus. Second, the SNPs employed by GWAS are common, but not rare, variants. Numerous studies have shown that many gene-disease associations are not due to a single common variant, but rather due to a constellation of more rare, regionally concentrated, disease-causing variants. Thus, the signals of association credited to our common SNPs may be synthetic associations resulting from the contributions of multiple rare SNPs within the *PHF3-PTP4A1* region, which need to be identified by sequencing. Third, both *PHF3* and *PTP4A1* were found to have significant association and functional signals. *PHF3* had weaker association signals in AAs and EAs and weaker functional signals in lymphoblastoid cell lines than *PTP4A1*. However, associations for *PHF3* markers were also replicated in the Australian sample. *PHF3* had greater evidence of altered RNA secondary structures than *PTP4A1*. These positive signals might be due to the LD with a single causal locus in *PHF3-PTP4A1* region, and this putative causal locus was more likely to be located in *PHF3* based on our current evidence, which, again, needs sequencing to confirm. Finally, HapMap JPT and YRI-Children populations also presented functional signals, but the distributions of −log(P) values across the LD block in these two populations were negatively correlated with those in AAs, EAs, Australians, HapMap CHB and CEU-Children. It is likely that, in these two sets of populations, different phases of alleles might be in LD with the same causal allele.

The Plant HomeoDomain (PHD) finger proteins (PHFs) are members of zinc finger protein (ZNF) superfamily. They are regulatory proteins in nucleus and cytoplasm and are frequently associated with chromatin-mediated transcriptional regulation [24], [25]. They can specifically recognize and bind to the tri-methylated lysines (e.g., H3K4me3 or H3K9me3) on histones, and regulate their methylation status. PHF3 is ubiquitously expressed in normal tissues including brain. It has been reported that alcohol abuse could significantly up-regulate the gene expression level of *PHF3* in the frontal cortex in alcoholics [26].

Additionally, the prenylated protein tyrosine phosphatases (PTPs) are cell signaling molecules that play regulatory roles in a variety of cellular processes. Over-expression of PTPs in mammalian cells confers a transformed phenotype, which implicates its role in diseases. It has been reported that, in mice, *Ptp4a1* expression was significantly regulated by ethanol in prefrontal cortex [27]; and transcript expression of *Ptp4a1* (p = 3.2×10^−11^) was significantly associated with alcohol consumption [28]. These findings supported *PHF3* and *PTP4A1* as reasonable candidates for alcohol dependence, although the biological mechanisms warrant more studies in the future.

## Supporting Information

Figure S1 **Manhattan plot for the p-values in AA case-control sample.** [*Y-axis: −log0.05 = 1.3; −log10^−5^ = 5; −log(5×10^−8^) = 7.3. X-axis: Chr1-22 = Autosomes; X = ChrX; Y = ChrY; X/Y = Pseudo-autosomal homologous regions of ChrX and ChrY; M = Mitochondrial chromosome; SNPs were ordered by physical distance within each chromosome/region*].
(TIF)

Table S1 **The 5 top-ranked SNPs associated with alcohol dependence in AA discovery sample.** [Genotypewise, allelewise: genotypewise and allelewise GWAS analysis. Before, after: association analysis before and after controlling for admixture effects, respectively].
(DOC)

Table S2 **P-values for associations of transcript expression between** ***PHF3-PTP4A1*** **and other genes in brain.**
(DOC)

Table S3 **P-values for the top-ranked SNPs with replicable associations between AAs and EAs.** [*Only the top-ranked SNPs that have p<9.9×10^−5^ for allelewise association analysis in AA discovery sample are listed. “Before”, “After”, before and after controlling for admixture effects, respectively*].
(DOC)

Table S4 **Bioinformatics of replicable risk SNPs in** ***PHF3-PTP4A1*** **.** [*These SNPs are located in the transcription factor-binding site; Bold SNPs can significantly (underlined; in PHF3) or slightly (in PTP4A1) alter the RNA secondary structures. Some databases categorize the SNPs in the 3′ flanking region of PHF3 (from rs319924 to rs3003672) into LOC389405 that encodes a notch 5-like protein similar to Neurogenic locus Notch protein precursor. OR, odds ratio directions corresponding to *Table 1* *: “−” denotes OR<1, “+” denotes OR>1. NA, not available.*]
(DOC)

Materials and Methods S1 (DOC)
