# Supplementary figures and images for: A Novel, Functional and Replicable Risk Gene Region for Alcohol Dependence Identified by Genome-Wide Association Study
Source: PLoS One. 2011 Nov 7;6(11):e26726. doi: 10.1371/journal.pone.0026726 (PMC3210123; doi:10.1371/journal.pone.0026726)

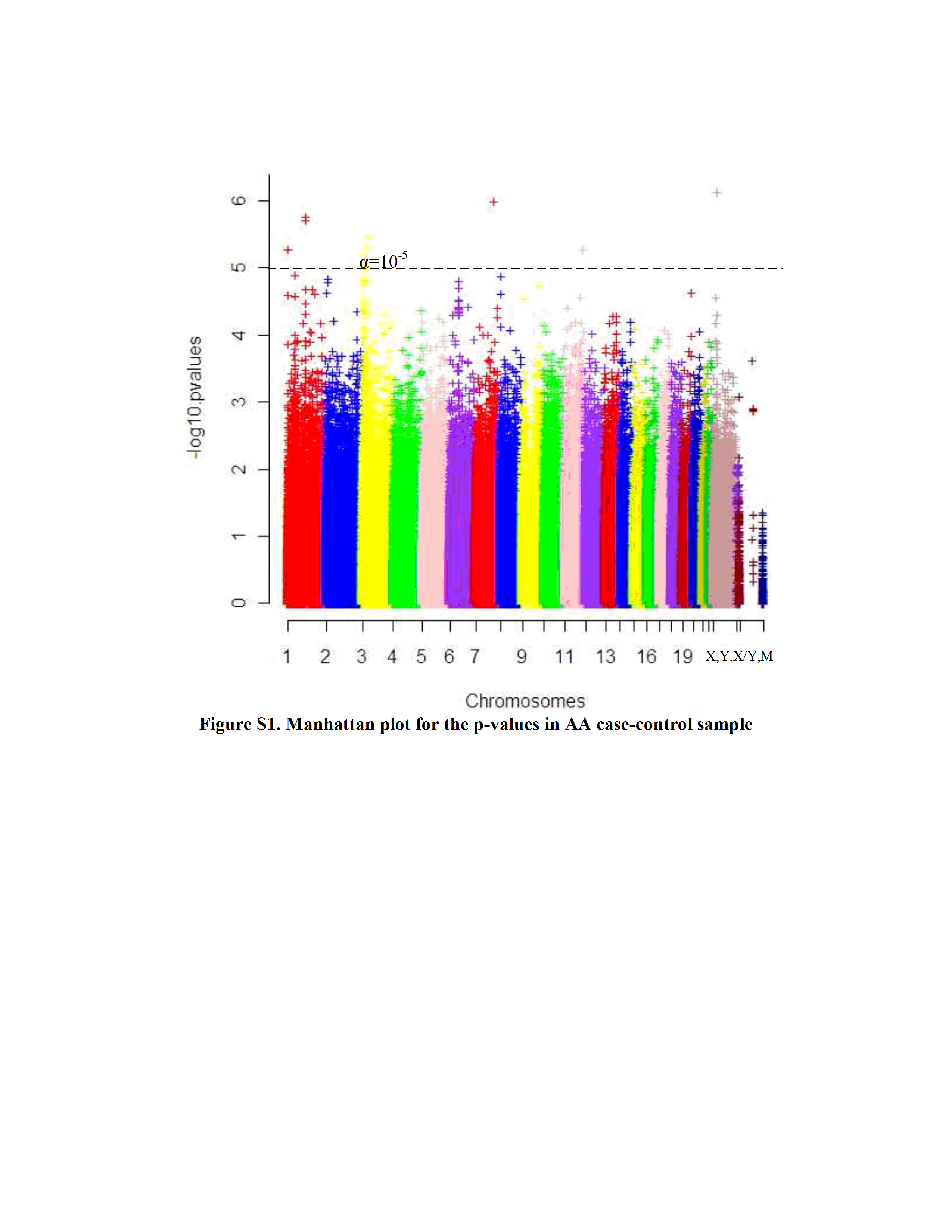

Supplement: Figure S1 — Manhattan plot for the p-values in AA case-control sample. [Y-axis: −log0.05 = 1.3; −log10−5 = 5; −log(5×10^−8^) = 7.3. X-axis: Chr1-22 = Autosomes; X = ChrX; Y = ChrY; X/Y = Pseudo-autosomal homologous regions of ChrX and ChrY; M = Mitochondrial chromosome; SNPs were ordered by physical distance within each chromosome/region]. (TIF) [file pone.0026726.s001.tif]
