# Supplementary material for: A Novel, Functional and Replicable Risk Gene Region for Alcohol Dependence Identified by Genome-Wide Association Study
Source: PLoS One. 2011 Nov 7;6(11):e26726. doi: 10.1371/journal.pone.0026726 (PMC3210123; doi:10.1371/journal.pone.0026726)
Supplement: Table S1 — The 5 top-ranked SNPs associated with alcohol dependence in AA discovery sample. [Genotypewise, allelewise: genotypewise and allelewise GWAS analysis. Before, after: association analysis before and after controlling for admixture effects, respectively]. (DOC) [file pone.0026726.s006.doc]

**Supplemental Table S1. The 5 top-ranked SNPs associated with alcohol dependence in AA discovery sample**

|  |  |  |  | p values | |
| --- | --- | --- | --- | --- | --- |
|  | SNPs | Chromosome | Gene/Locus | Before | After |
| Genotypewise | rs11922615 | 3p25.3 | LOC440944 | 2.6×10^-8^ | 1.9×10^-7^ |
| Genotypewise | rs6443238 | 3p25.3 | LOC440944 | 5.1×10^-8^ | 4.4×10^-7^ |
| Genotypewise | rs7777391 | 7q31 | CTTNBP2 | 3.0×10^-7^ | 7.0×10^-7^ |
| Genotypewise | rs3792686 | 4p16.3 | D4S234E | 5.8×10^-7^ | 1.7×10^-6^ |
| Genotypewise | rs17028719 | 1p36.22 | NPHP4 | 8.9×10^-7^ | 4.2×10^-6^ |
| Allelewise | rs4610908 | Xp21.1 | FAM47B | 5.7×10^-7^ | 7.5×10^-7^ |
| Allelewise | rs7777391 | 7q31 | CTTNBP2 | 7.1×10^-7^ | 1.0×10^-6^ |
| Allelewise | rs699771 | 1p13.3-13.1 | HAO2 | 4.0×10^-6^ | 1.7×10^-6^ |
| Allelewise | rs1891224 | 1p13.3-13.1 | HAO2 | 4.2×10^-6^ | 1.9×10^-6^ |
| Allelewise | rs10446425 | 3p21 | CTNNB1 | 1.2×10^-5^ | 3.4×10^-6^ |

Genotypewise, allelewise: genotypewise and allelewise GWAS analysis. Before, after: association analysis before and after controlling for admixture effects, respectively.
