# Supplementary material for: A Novel, Functional and Replicable Risk Gene Region for Alcohol Dependence Identified by Genome-Wide Association Study
Source: PLoS One. 2011 Nov 7;6(11):e26726. doi: 10.1371/journal.pone.0026726 (PMC3210123; doi:10.1371/journal.pone.0026726)
Supplement: Table S2 — P-values for associations of transcript expression between PHF3-PTP4A1 and other genes in brain. (DOC) [file pone.0026726.s002.doc]

**Supplemental Table S2. P-values for correlations of transcript expression between *PHF3-PTP4A1* and other genes in brain**

| Genes | PHF3 | PTP4A1 |
| --- | --- | --- |
| MTHFR | 1.5×10^-11^ | 1.8×10^-7^ |
| NRD1 | <2×10^-16^ | <2×10^-16^ |
| PDE4B | 1.6×10^-12^ | 9.7×10^-15^ |
| OLFM3 |  | 8.0×10^-10^ |
| HNMT | 2.8×10^-13^ | 8.6×10^-15^ |
| NXPH2 | 2.1×10^-7^ |  |
| GAD1 |  | 3.3×10^-11^ |
| PECR | 3.9×10^-15^ | 8.4×10^-15^ |
| PPARG | 7.6×10^-7^ | 3.9×10^-13^ |
| SH3BP5 | 4.7×10^-14^ | <2×10^-16^ |
| BBX | <2×10^-16^ | 2.5×10^-10^ |
| PCDH7 |  | 4.7×10^-8^ |
| GABRA2 |  | 6.9×10^-9^ |
| GABRB1 |  | 4.3×10^-9^ |
| SNCA |  | 3.1×10^-10^ |
| LOC91431 | <2×10^-16^ | 2.5×10^-12^ |
| IPO11 | 8.0×10^-14^ | <2×10^-16^ |
| CAST | <2×10^-16^ | 6.7×10^-14^ |
| ERAP1 | <2×10^-16^ | 9.3×10^-16^ |
| PPP2R2B | 3.5×10^-10^ | <2×10^-16^ |
| GABRB2 |  | 2.1×10^-7^ |
| GABRA1 |  | 4.5×10^-11^ |
| GABRG2 |  | 1.9×10^-10^ |
| FAM44B | <2×10^-16^ | <2×10^-16^ |
| ANKS1A | 1.5×10^-8^ |  |
| CNR1 |  | 1.7×10^-7^ |
| EPHA7 |  | 1.5×10^-11^ |
| FYN | 1.2×10^-13^ | 2.1×10^-9^ |
| DRD4 | 9.3×10^-9^ | 7.6×10^-11^ |
| TH | 4.3×10^-7^ | 3.1×10^-8^ |
| NAP1L4 | 3.3×10^-14^ |  |
| CARS | 3.3×10^-7^ | 4.7×10^-7^ |
| CAT | 1.3×10^-10^ |  |
| API5 | 4.8×10^-10^ | 1.3×10^-15^ |
| DRD2 | 2.8×10^-11^ | 1.8×10^-13^ |
| HTR3B |  | 1.2×10^-9^ |
| CCDC41 | 1.6×10^-13^ | 2.2×10^-13^ |
| HTR2A |  | 1.5×10^-10^ |
| PCDH9 | 3.1×10^-13^ | 3.5×10^-9^ |
| CDH8 |  | 2.6×10^-10^ |
| CDH13 |  | 8.5×10^-10^ |
| SLC6A4 |  | 1.8×10^-7^ |
