# Supplementary material for: A Novel, Functional and Replicable Risk Gene Region for Alcohol Dependence Identified by Genome-Wide Association Study
Source: PLoS One. 2011 Nov 7;6(11):e26726. doi: 10.1371/journal.pone.0026726 (PMC3210123; doi:10.1371/journal.pone.0026726)
Supplement: Table S3 — P-values for the top-ranked SNPs with replicable associations between AAs and EAs. [Only the top-ranked SNPs that have p<9.9×10^−5^ for allelewise association analysis in AA discovery sample are listed. “Before”, “After”, before and after controlling for admixture effects, respectively]. (DOC) [file pone.0026726.s003.doc]

**Supplemental Table S3. P-values for the top-ranked SNPs with replicable associations between AAs and EAs**

|  |  |  | African-American (AA) discovery sample | | | | | European-American (EA) replication sample | | | | |
| --- | --- | --- | --- | --- | --- | --- | --- | --- | --- | --- | --- | --- |
|  |  |  | Allelewise . | | | Genotypewise . | | Allelewise . | | | Genotypewise | |
| CHR | Gene | SNP | OR | Before | After | Before | After | OR | Before | After | Before | After |
| 3p25.3 | SLC6A11 | rs3774084 | 2.27 | 5.7×10^-5^ | 6.5×10^-6^ | 8.3×10^-5^ | 3.5×10^-5^ | 1.11 | 0.097 | 0.049 | >0.05 | 0.127 |
| 6q12 | PHF3 | rs9294269 | 1.56 | 3.7×10^-6^ | 1.6×10^-5^ | 2.3×10^-5^ | 9.2×10^-5^ | 0.85 | 4.1×10^-3^ | 2.4×10^-3^ | 2.8×10^-3^ | 2.0×10^-3^ |
| 6q12 | PTP4A1 | rs6942342 | 1.56 | 4.1×10^-6^ | 2.0×10^-5^ | 2.6×10^-5^ | 1.2×10^-4^ | 0.85 | 4.5×10^-3^ | 1.8×10^-3^ | 9.4×10^-3^ | 5.2×10^-3^ |
| 6q12 | PTP4A1 | rs1322416 | 1.54 | 7.5×10^-6^ | 3.0×10^-5^ | 5.0×10^-5^ | 1.7×10^-4^ | 0.86 | 0.011 | 5.2×10^-3^ | 0.011 | 7.0×10^-3^ |
| 6q12 | PTP4A1 | rs4299811 | 1.54 | 7.0×10^-6^ | 3.0×10^-5^ | 4.7×10^-5^ | 1.7×10^-4^ | 0.85 | 5.0×10^-3^ | 2.5×10^-3^ | 0.011 | 6.5×10^-3^ |
| 6q12 | PTP4A1 | rs9449291 | 1.54 | 7.8×10^-6^ | 3.8×10^-5^ | 4.4×10^-5^ | 2.0×10^-4^ | 0.85 | 6.7×10^-3^ | 3.1×10^-3^ | 0.016 | 9.9×10^-3^ |
| 6q12 | PTP4A1 | rs1681957 | 1.54 | 9.1×10^-6^ | 3.8×10^-5^ | 6.2×10^-5^ | 2.1×10^-4^ | 0.85 | 7.3×10^-3^ | 3.3×10^-3^ | 0.012 | 7.3×10^-3^ |
| 6q12 | PTP4A1 | rs1197905 | 1.54 | 9.1×10^-6^ | 3.8×10^-5^ | 6.2×10^-5^ | 2.1×10^-4^ | 0.85 | 6.7×10^-3^ | 3.0×10^-3^ | 0.010 | 6.0×10^-3^ |
| 6q12 | PTP4A1 | rs9353016 | 1.54 | 1.0×10^-5^ | 4.1×10^-5^ | 6.1×10^-5^ | 2.3×10^-4^ | 0.85 | 4.9×10^-3^ | 2.2×10^-3^ | 8.9×10^-3^ | 5.2×10^-3^ |
| 6q12 | PTP4A1 | rs2984458 | 1.52 | 1.1×10^-5^ | 4.4×10^-5^ | 7.0×10^-5^ | 2.4×10^-4^ | 0.85 | 6.7×10^-3^ | 3.0×10^-3^ | 0.011 | 6.5×10^-3^ |
| 6q12 | PTP4A1 | rs4557499 | 1.52 | 1.0×10^-5^ | 4.5×10^-5^ | 6.5×10^-5^ | 2.5×10^-4^ | 0.85 | 4.9×10^-3^ | 2.2×10^-3^ | 9.6×10^-3^ | 5.6×10^-3^ |
| 6q12 | PTP4A1 | rs1744140 | 1.52 | 1.2×10^-5^ | 4.9×10^-5^ | 8.0×10^-5^ | 2.7×10^-4^ | 0.85 | 6.7×10^-3^ | 3.0×10^-3^ | 0.012 | 6.8×10^-3^ |
| 6q12 | PTP4A1 | rs2758259 | 1.52 | 1.1×10^-5^ | 5.0×10^-5^ | 7.1×10^-5^ | 2.8×10^-4^ | 0.85 | 4.4×10^-3^ | 1.8×10^-3^ | 8.3×10^-3^ | 4.5×10^-3^ |
| 1p34 | APOER2 | rs1416095 | 0.60 | 7.2×10^-5^ | 1.2×10^-5^ | 2.7×10^-4^ | 6.6×10^-5^ | 1.17 | 4.2×10^-3^ | 5.4×10^-3^ | 4.4×10^-4^ | 7.9×10^-4^ |
| 1p34 | APOER2 | rs3820198 | 0.61 | 1.2×10^-4^ | 2.7×10^-5^ | 5.0×10^-4^ | 1.4×10^-4^ | 1.15 | 0.012 | 0.015 | 2.6×10^-3^ | 4.1×10^-3^ |
| 4q35.2 | ZNF754 | rs7683009 | 0.68 | 1.1×10^-4^ | 4.2×10^-5^ | 1.8×10^-4^ | 9.8×10^-5^ | 1.11 | 0.034 | 0.041 | 0.077 | 0.100 |
| 2p16 | EFEMP1 | rs17047868 | 1.73 | 8.7×10^-5^ | 6.0×10^-5^ | 3.4×10^-4^ | 2.4×10^-4^ | 4.17 | 0.082 | 0.040 | 0.082 | 0.040 |
| 1q42 | TAF1A | rs2936032 | 1.45 | 5.4×10^-5^ | 6.6×10^-5^ | 3.4×10^-4^ | 2.6×10^-4^ | 1.25 | 0.016 | 3.6×10^-3^ | 0.057 | 0.014 |
| 1p21 | EDG1 | rs17417367 | 0.38 | 1.1×10^-4^ | 6.7×10^-5^ | 1.2×10^-4^ | 5.9×10^-4^ | 0.86 | 0.087 | 0.072 | >0.05 | 0.134 |
| 8q13.1 | SULF1 | rs16936012 | 1.71 | 7.3×10^-5^ | 8.4×10^-5^ | 3.1×10^-4^ | 5.1×10^-4^ | 1.80 | 0.015 | 2.4×10^-3^ | 0.040 | 0.010 |
| 14q32.11 | KCNK13 | rs7149330 | 0.72 | 3.5×10^-4^ | 8.5×10^-5^ | 2.0×10^-3^ | 4.4×10^-4^ | 1.14 | 0.069 | 0.065 | >0.05 | 0.148 |
| 17q22-23.2 | PRKCA | rs8081614 | 1.46 | 1.6×10^-4^ | 9.3×10^-5^ | 3.4×10^-4^ | 3.5×10^-4^ | 1.14 | 0.091 | 0.047 | >0.05 | 0.130 |

*Only the top-ranked SNPs that have p<9.9×10^-5^ for allelewise association analysis in AA discovery sample are listed. “Before”, “After”, before and after controlling for admixture effects, respectively.*
