# Supplementary material for: A Novel, Functional and Replicable Risk Gene Region for Alcohol Dependence Identified by Genome-Wide Association Study
Source: PLoS One. 2011 Nov 7;6(11):e26726. doi: 10.1371/journal.pone.0026726 (PMC3210123; doi:10.1371/journal.pone.0026726)
Supplement: Table S4 — Bioinformatics of replicable risk SNPs in PHF3-PTP4A1 . [*These SNPs are located in the transcription factor-binding site; Bold SNPs can significantly (underlined; in PHF3) or slightly (in PTP4A1) alter the RNA secondary structures. Some databases categorize the SNPs in the 3′ flanking region of PHF3 (from rs319924 to rs3003672) into LOC389405 that encodes a notch 5-like protein similar to Neurogenic locus Notch protein precursor. OR, odds ratio directions corresponding to Table 1 : “−” denotes OR<1, “+” denotes OR>1. NA, not available.] (DOC) [file pone.0026726.s004.doc]

**Supplemental Table S4. Bioinformatics of replicable risk SNPs in *PHF3-PTP4A1***

|  |  | CHR |  | Nearby | Regulatory | Conservation | Minor |  | Allele frequencies . | | | |
| --- | --- | --- | --- | --- | --- | --- | --- | --- | --- | --- | --- | --- |
| SNP | Gene | Position | Location | Genes | Potential Score | Score | Allele | OR | AAs | YRI | EAs | CEU |
| rs9449291 | PTP4A1 | 64220912 | flanking_5’-UTR | GLULD1||PHF3 | 0.000 | 0.001 | A | + | 0.175 | 0.210 | 0.565 | 0.504 |
| rs9449312 | PTP4A1 | 64227493 | flanking_5’-UTR | GLULD1||PHF3 | NA | 0.000 | C | + | 0.436 | 0.527 | 0.574 | 0.527 |
| rs6942342 | PTP4A1 | 64234406 | flanking_5’-UTR | GLULD1||PHF3 | 0.000 | 0.001 | C | + | 0.174 | 0.217 | 0.571 | 0.518 |
| rs9353016***** | PTP4A1 | 64243991 | flanking_5’-UTR | GLULD1||PHF3 | 0.086 | 0.996 | A | + | 0.177 | 0.217 | 0.570 | 0.514 |
| rs4299811***** | PTP4A1 | 64248405 | flanking_5’-UTR | GLULD1||PHF3 | 0.038 | 0.380 | T | + | 0.173 | 0.236 | 0.570 | 0.518 |
| rs4557499***** | PTP4A1 | 64248506 | flanking_5’-UTR | GLULD1||PHF3 | NA | 0.001 | T | + | 0.176 | 0.217 | 0.570 | 0.513 |
| **rs2758259** | PTP4A1 | 64251066 | flanking_5’-UTR | GLULD1||PHF3 | NA | 0.000 | C | + | 0.177 | 0.217 | 0.569 | 0.513 |
| **rs1744134** | PTP4A1 | 64262673 | flanking_5’-UTR | GLULD1||PHF3 | 0.000 | 0.060 | G | - | 0.287 | 0.204 | 0.347 | 0.392 |
| rs1744140 | PTP4A1 | 64270790 | flanking_5’-UTR | GLULD1||PHF3 | 0.000 | 0.001 | T | + | 0.179 | 0.217 | 0.570 | 0.513 |
| **rs2984458** | PTP4A1 | 64273122 | flanking_5’-UTR | GLULD1||PHF3 | NA | 0.000 | T | + | 0.179 | 0.217 | 0.569 | 0.513 |
| rs1681957 | PTP4A1 | 64281104 | flanking_5’-UTR | GLULD1||PHF3 | NA | 0.001 | T | + | 0.178 | 0.217 | 0.569 | 0.513 |
| rs1197905 | PTP4A1 | 64292965 | flanking_5’-UTR | GLULD1||PHF3 | 0.000 | 0.005 | T | + | 0.178 | 0.217 | 0.570 | 0.518 |
| rs2622274 | PTP4A1 | 64298475 | flanking_5’-UTR | GLULD1||PHF3 | 0.000 | 0.000 | G | + | 0.411 | 0.491 | 0.571 | 0.518 |
| rs1322416 | PTP4A1 | 64311978 | flanking_5’-UTR | GLULD1||PHF3 | NA | 0.000 | T | + | 0.179 | 0.212 | 0.568 | 0.523 |
| rs9294269 | PHF3 | 64395256 | flanking_5’-UTR | PTP4A1||EGFL10 | NA | 0.010 | C | + | 0.177 | 0.212 | 0.571 | 0.518 |
| rs6932538 | PHF3 | 64440939 | intron 1 | PTP4A1||EGFL10 | NA | 0.000 | C | + | 0.202 | 0.208 | 0.555 | 0.491 |
| **rs10485358** | PHF3 | 64444019 | intron 1 | PTP4A1||EGFL10 | 0.000 | 0.001 | C | + | 0.176 | 0.19 | 0.553 | 0.482 |
| **rs10755432** | PHF3 | 64455812 | intron 3 | PTP4A1||EGFL10 | NA | 0.188 | A | + | 0.192 | 0.204 | 0.553 | 0.487 |
| **rs1057530** | PHF3 | 64485054 | flanking_3’-UTR | PTP4A1||EGFL10 | 0.000 | 0.003 | C | + | 0.234 | 0.221 | 0.556 | 0.496 |
| **rs12205302** | PHF3 | 64496875 | flanking_3’-UTR | PTP4A1||EGFL10 | NA | 0.000 | G | + | 0.227 | 0.221 | 0.558 | 0.491 |
| **rs319924** | PHF3 | 64545206 | flanking_3’-UTR | PTP4A1||EGFL10 | 0.000 | 0.000 | A | + | 0.208 | 0.208 | 0.616 | 0.554 |
| rs319920 | PHF3 | 64562211 | flanking_3’-UTR | PTP4A1||EGFL10 | 0.000 | 0.000 | A | + | 0.204 | 0.208 | 0.615 | 0.554 |
| rs756274 | PHF3 | 64563172 | flanking_3’-UTR | PTP4A1||EGFL10 | 0.000 | 0.001 | A | + | 0.444 | 0.447 | 0.647 | 0.612 |
| **rs6921058** | PHF3 | 64582110 | flanking_3’-UTR | PTP4A1||EGFL10 | 0.000 | 0.001 | G | + | 0.230 | 0.221 | 0.615 | 0.549 |
| **rs12205984** | PHF3 | 64630578 | flanking_3’-UTR | PTP4A1||EGFL10 | 0.000 | 0.000 | A | + | 0.277 | 0.292 | 0.612 | 0.540 |
| **rs321498** | PHF3 | 64637183 | flanking_3’-UTR | PTP4A1||EGFL10 | 0.000 | 0.000 | C | + | 0.408 | 0.509 | 0.687 | 0.625 |
| rs321494 | PHF3 | 64637766 | flanking_3’-UTR | PTP4A1||EGFL10 | 0.141 | 0.000 | T | + | 0.214 | 0.226 | 0.610 | 0.540 |
| rs729291 | PHF3 | 64653033 | flanking_3’-UTR | PTP4A1||EGFL10 | 0.000 | 0.000 | T | + | 0.272 | 0.292 | 0.609 | 0.545 |
| **rs1482451** | PHF3 | 64655045 | flanking_3’-UTR | PTP4A1||EGFL10 | 0.000 | 0.000 | A | - | 0.392 | 0.311 | 0.297 | 0.348 |
| **rs3003672** | PHF3 | 64889079 | flanking_3’-UTR | PTP4A1||EGFL10 | 0.000 | 0.000 | C | - | 0.291 | 0.398 | 0.003 | 0.003 |

**These SNPs are located in the transcription factor-binding site; Bold SNPs can significantly (underlined; in PHF3) or slightly (in PTP4A1) alter the RNA secondary structures. Some databases categorize the SNPs in the 3’ flanking region of PHF3 (from rs319924 to rs3003672) into LOC389405 that encodes a notch 5-like protein similar to Neurogenic locus Notch protein precursor. OR, odds ratio directions in AAs corresponding to Table 1: “-“ denotes OR<1, “+” denotes OR>1. NA, not available.*
