# Supplementary material for: A Novel, Functional and Replicable Risk Gene Region for Alcohol Dependence Identified by Genome-Wide Association Study
Source: PLoS One. 2011 Nov 7;6(11):e26726. doi: 10.1371/journal.pone.0026726 (PMC3210123; doi:10.1371/journal.pone.0026726)
Supplement: Materials and Methods S1 — (DOC) [file pone.0026726.s005.doc]

**Supplemental Materials and Methods S1**

**Subjects: Unrelated American samples:** A total of 4,316 SAGE (dbGaP study accession phs000092.v1.p1) subjects and 1,957 COGA (dbGaP study accession phs000125.v1.p1) subjects were merged into a single dataset. 1,445 subjects in COGA that overlapped with SAGE were excluded. SAGE subjects were recruited from 8 different study sites in 7 states and the District of Columbia; the majority of subjects (62%) were recruited in Missouri [1]. COGA subjects were recruited from 7 centers in the United States [2]. All subjects were interviewed using the Semi-Structured Assessment for the Genetics of Alcoholism (SSAGA) [3]. Affected subjects were excluded if they had schizophrenia or other psychotic illnesses (n=314). These alcohol dependent subjects had comorbid marijuana dependence (34.8%), opioid dependence (13.8%), nicotine dependence (61.0%), cocaine dependence (48.2%) or other illicit substance (stimulants, sedatives) dependence (5.8%). **Australian family samples:** A total of 6,701 OZ-ALC (the Australian family study of alcohol use disorder; dbGaP study accession phs000181.v1.p1) subjects including twins and their parents, siblings, spouses, children and other family members were included in the dataset. The index cases either reported a history of alcohol dependence (DSM-IV), or scored above the 85th percentile on a quantitative measure of heaviness of alcohol use (alcohol factor score), derived from measures of frequency of heavy drinking, frequency of drinking to intoxication, and typical weekly consumption in standard drinks and of lifetime maximum 1-day alcohol consumption and maximum tolerance to alcohol. The demographic data of SAGE, COGA and OZ-ALC subjects have been presented previously [1,2,4].

**Genotyping platform:** All samples were genotyped on the Illumina Human 1M (for SAGE and COGA) or CNV370v1 (for OZ-ALC) beadchips at the Center for Inherited Disease Research (CIDR) at Johns Hopkins University (Baltimore, MD USA) or at deCODE (Reykjavik, Iceland). Allele cluster definitions for each marker were determined using Illumina BeadStudio Genotyping Module version 3.1.14 and the combined intensity data from the samples.

**Data analytic procedure summary:** In the present study, the AA case-control sample served as the discovery sample; an independent EA case-control sample served as the primary replication sample; and an independent Australian family sample served as the secondary replication sample. Genome-wide association analysis was first performed in the discovery sample, to derive the highest risk SNPs with p<10^-4^ (termed “top-ranked SNPs” in the text). Associations of all top-ranked SNPs in AAs were retested in EAs, to detect replicable risk SNPs. Because the gene that contained more replicable risk top-ranked SNPs (i.e., *PHF3-PTP4A1* locus in this study) may have higher probability to harbor disease-causing loci, it may contain more other replicable but less significant risk SNPs in LD with the putative disease loci. Therefore, all nominally significant SNPs (p<0.05) within this locus in AAs were also retested in the EA sample to detect these replicable risk SNPs; and then associations for all replicable risk SNPs were also retested in the Australian family sample (**Table 1**).

In addition to the replication design for the gene-disease associations between AAs, EAs and Australians, we also performed functional analyses on the replicable risk SNPs as confirmation design. These analyses included (a) *cis-*acting expression quantitative trait locus (*cis-*eQTL) analysis on mRNA expression levels in lymphoblastoid cell lines, and (b) alteration of RNA secondary structure by these replicable risk variants. Furthermore, correlations of transcript expression in brain between the risk genes and other genes across the transcriptome were analyzed. Finally, correlations on the distributions of -log(p) values across AAs, EAs, Australians and six HapMap populations were tested.

**Rationale of replication strategy:** Allele frequencies could be different in distinct populations, even in opposite phases, i.e., a common allele in one population may be a rare allele in another population. Thus, distinct populations do not necessarily have the same risk markers associated with disease; alternatively, they could have the same risk markers, but different phases of alleles in these markers are associated with disease. That is, the effect sizes and effect directions of marker-disease associations may be not consistent, even opposite between distinct populations for each individual risk marker, so that meta-analysis may show weaker effects. Such markers were treated as non-replicable ones previously and thus were discarded. However, to our hypothesis, when two distinct populations have common causal variants, there could be a risk region in LD with this putative causal variant in both populations, even though there are no individual risk alleles replicable between them. This is because in one population, a set of risk markers are in LD with the causal variant; but in another population, a different set of risk markers adjacent to the first set could be in LD with the causal variant. The risk marker sets in a same causal region are different between populations, which is because they are not causal variants *per se*. Such a risk region may have a significant correlation between the distributions of -log(p) values of all markers across whole region in different populations. Such regions were missed in previous studies. Additionally, in the present study, the datasets for association studies and for eQTL analysis were different in many absolute statistics of genetic marker numbers, sample sizes and study power that affected the absolute values of p. To study the consistency between them, we can only compare their relative statistics, i.e., the distributions of relative significance strengths (e.g., -log(p)) across whole region, not individual markers. The correlation of distributions of -log(p) values between two samples was a relative statistic that was not influenced by the study power (including the sample sizes) and thus was appropriate to be used to evaluate the consistency of association signals between two samples.

We used functional analysis to the follow-up association findings, based on the hypothesis that only functional SNPs can cause disease. If a SNP is functional, the possibility of association between this SNP and disease to be true will significantly increase. We hypothesized that genetic markers with replicable association were highly likely to be functional, and those markers with more evidence of replicable association were more likely to be functional, and thus could be replicated in more samples and would be positive in functional analysis. This hypothesis was tested in the present study.

**References**

1. Bierut LJ, Agrawal A, Bucholz KK, Doheny KF, Laurie C, et al. (2010) A genome-wide association study of alcohol dependence. Proc Natl Acad Sci U S A 107: 5082-5087.

2. Edenberg HJ, Koller DL, Xuei X, Wetherill L, McClintick JN, et al. (2010) Genome-wide association study of alcohol dependence implicates a region on chromosome 11. Alcohol Clin Exp Res 34: 840-852.

3. Bucholz KK, Cadoret R, Cloninger CR, Dinwiddie SH, Hesselbrock VM, et al. (1994) A new, semi-structured psychiatric interview for use in genetic linkage studies: a report on the reliability of the SSAGA. J Stud Alcohol 55: 149-158.

4. Heath AC, Whitfield JB, Martin NG, Pergadia ML, Goate AM, et al. (2011) A quantitative-trait genome-wide association study of alcoholism risk in the community: findings and implications. Biol Psychiatry 70: 513-518.
